# The Classics of Immunology

**DOI:** 10.1186/1476-9433-4-3

**Published:** 2005-03-10

**Authors:** Kendall A Smith

**Affiliations:** 1 The Division of Immunology Department of Medicine Weill Medical College Cornell University New York, NY 10021 USA

## Abstract

Medical Immunology will be publishing invited Reviews and Commentaries from investigators who are at the forefront of their fields, to up-date our readers as to the current state of their art. These Reviews and Commentaries will be accompanied by Editorials that place the current work into the perspective of the first contribution in an area, which resulted in a "Classic" paper. Where possible, links will be provided to the original publication, so that the modern student of immunology can read the original and draw their own conclusions as to the value of the "Classic" contribution, and its relationship to our contemporary views as to how the immune system functions. To begin this process at the very dawn of immunology, we highlight Sir Edward Jenner's first descriptions of the use of cowpox to immunize individuals against the dread disease smallpox.

## Editorial

With this editorial, Medical Immunology embarks on a new endeavor. One of the advantages of the electronic/digital age is that so much of the scientific literature is now available to everyone, not only to those with access to the specialized scientific or medical libraries. However, unless one knows which author/investigator/topic to seek out, it is still difficult for the student or layperson to know where to begin and which key words to use in the search engines. Accordingly, Medical Immunology now is devoted to an historical development of the field of immunology, much of which was organized chronologically in my first editorial[1], which attempted to justify why we needed a new on-line journal devoted to Medical Immunology. So much of the more recent scientific literature is difficult for one who is uninitiated in the science to be able to just pick up and read outright, because each contribution usually describes only a segment of a larger work by the authors and many others, and each individual contribution is but a very small part of a much larger area of investigation. As well, if one is not schooled in the vocabulary, it is difficult, if not impossible to begin to understand experiments performed and the significance of the findings reported.

I have developed a method of inquiry of the scientific literature when embarking on a new subject that works for me, and allows me to organize the experimental findings and to rank them in importance one with another. Thus, I take a chronological approach, beginning with the very first report in a particular field, and then tracing the papers forward in time. This approach has the advantage of starting way in the past, at a time when often the concepts and methods of investigation were quite simple and therefore more easily understood, so that as one studies more recent reports it is much easier to appreciate the significance of a new approach or fresh insight into an old problem.

Therefore, with this as background, we begin our course in immunology with Sir Edward Jenner's ***"An Inquiry into the Causes and Effects of the Variolae Vaccinae, a Disease Discovered in some of the Western Counties of England, particularly Goucestershire, and known by the name of The Cow Pox"***, which was published in 1798 [2].

Reading through this detailed account of Jenner's experiments, which he conducted over several years, one is struck by the meticulous care this country doctor made in documenting his efforts, as well as his powers of observation, which allowed him to identify the unique characteristics of the cowpox lesions, so that he could transfer only the liquid from cowpox pustules from one individual to another. Of course, unbeknownst to Jenner at the time were the myriad of microorganisms that could mimic such pustules, such as the pyogenic bacteria such as staphylococci and streptococci. If he had not been so careful to detail and identify the characteristics of the cowpox lesions, he could have missed the observations that started the science of immunology, and that led to his worldwide fame and his Knighthood, as well as his lifetime pension from the Crown.

It is perhaps ironic that we find ourselves 207 years later still concerned with smallpox, and concerned because of the idea that this devastating organism could again be used as a weapon of biological warfare. The stories of the English soldiers, who gave smallpox-infected blankets to the North American Indians during the French and Indian Wars, are familiar to us. Now we are afraid that ***new ***foreign terrorists may do the same to us!

Even though Jenner's experiments showed us the way to effective vaccination, and even though smallpox was declared eradicated from the world in 1980, we still have not understood how to make an effective and safe vaccine. All of the vaccines that we have, including those developed empirically during the 1950's and 1960's, were developed before our present understanding of how the immune system functions, which has occurred only since 1970.

Unfortunately, because of the threat of bioterrorism, we are now hard-pressed to invent, as rapidly as possible, new vaccines to protect our population against smallpox, as well as many of the very pathogenic microorganisms for which we do not have safe and effective vaccines.

Accordingly, the questions as to how to construct new vaccines for smallpox, as well as many other infectious diseases are paramount. In this regard, how to test for the efficacy of vaccines is central to both the problem and the solutions. Of course, Jenner monitored a successful "take" of the cowpox inoculation as the development of a localized lesion that resembled the cowpox lesions on the hands of the milkmaids, and that resembled the skin lesions that became generalized after a smallpox infection. Accordingly, because the cowpox virus actually replicates in humans, it generated a localized self-limiting infection that could not only be detected with the naked eye, but by so doing, a very strong immunological stimulus resulted, which left the host with long-lasting immunological memory.

Now that we know much more than did Jenner more than 200 years ago about how the immune system functions, one might imagine that it should be relatively easy to construct a safe and effective smallpox vaccine. Therefore, to begin our discussions in this regard, I have asked Mark Slifka, from the Vaccine and Gene Therapy Institute at the Oregon Health & Sciences University, to write a Commentary on the current state of the art of immunity to smallpox that can be detected by our most modern methods of monitoring the immune response, and to identify where we are, and where we need to go. Mark is especially suited to educate us, in that his group has published one of the most extensive analyses of the immunologic memory of individuals vaccinated against smallpox, using modern up-to-date methods [3].

This effort in *Classics in Immunology *should be construed as an open invitation for investigators to send us contributions that deal with new approaches to smallpox vaccines, as well as the use of smallpox vaccines as vectors for other microbes. As well, it is an invitation to all readers to write to the editors with questions or comments.
